# Assessment of Mental Health Services Available Through Smartphone Apps

**DOI:** 10.1001/jamanetworkopen.2022.48784

**Published:** 2022-12-28

**Authors:** Erica Camacho, Asher Cohen, John Torous

**Affiliations:** 1 Department of Psychiatry, Beth Israel Deaconess Medical Center, Boston, Massachusetts

## Abstract

**Importance:**

As more patients and clinicians are turning to mental health smartphone apps to expand access to services, little is known about the current state of the app marketplaces and what these apps are actually offering in terms of features, privacy, price, and services.

**Objective:**

To assess the current state of mental health apps, explore the association between app privacy scores and popularity as measured by star ratings and downloads, and to understand opportunities and challenges facing the commercial app landscape.

**Design, Setting, and Participants:**

This cross-sectional study had trained raters using the public-facing M-Health Index and Navigation Database (MIND) to assess and review 578 mental health apps. The sample of apps used in this analysis were pulled from MIND and include apps across various conditions including schizophrenia, eating disorders, sleep, and more. Analysis of these apps was conducted in June 2022.

**Exposures:**

There were 578 mental health apps rated across 105 dimensions derived from the American Psychiatric Association’s app evaluation framework.

**Main Outcomes and Measures:**

App raters assessed each app across 6 categories: (1) app origin and accessibility, (2) privacy and security, (3) clinical foundation, (4) features and engagement, (5) inputs and outputs, and (6) interoperability. Privacy scores were determined by 5 MIND criteria, including (1) having a privacy policy, (2) reporting security measures in place, (3) declaring data use and purpose, (4) allowing for the deletion of data, and (5) allowing users to opt out of data collection. Correlations between privacy scores and popularity metrics (star ratings and number of downloads) were measured.

**Results:**

This study included 578 mental health apps that were identified, assessed, and analyzed across 105 MIND dimensions. Psychoeducation, goal setting, and mindfulness were among the top app features. Of the 578 apps analyzed, 443 (77%) had a privacy policy. This analysis of apps with a privacy policy revealed that there was no statistically significant correlation between privacy scores and Apple App Store (*r* = 0.058, *P* = .29) or Google Play Store star ratings (*r* = 0.041; *P* = .48). The number of app downloads on the Google Play Store, however, was weakly correlated with privacy scores (χ^2^_5_ = 22.1; *P* < .001).

**Conclusions and Relevance:**

In this cross-sectional study of mental health apps, findings indicate that the current app marketplaces primarily offered basic features such as psychoeducation, goal tracking, and mindfulness but fewer innovative features such as biofeedback or specialized therapies. Privacy challenges remained common, and app popularity metrics provided little help in identifying apps with more privacy.

## Key Points

**Question:**

What do mental health smartphone apps offer patients, how has the app landscape changed, and are app popularity metrics associated with privacy?

**Findings:**

In this cross-sectional study of 578 mental health apps, an app marketplace assessment found that while more apps were collecting passive data, most apps still offered similar foundational features. There was no statistically significant correlation between privacy scores and star ratings, but there was a weak correlation between privacy scores and app downloads.

**Meaning:**

These findings suggest that apps on the marketplace offer overlapping features, and metrics such as star ratings or the number of downloads may not provide adequate information about the privacy or efficacy of mental health apps.

## Teaser

This cross-sectional study assesses the current state of mental health smartphone apps and the association between app privacy scores and popularity as measured by star ratings and downloads.

## Introduction

COVID-19 has increased demands on the mental health care system and led to increased reliance on digital tools for care delivery.^1^ While synchronous telehealth (eg, video visits) has now become commonplace, asynchronous tools such as smartphone apps have also expanded in popularity. With more than 10 000 mental health–related apps on the marketplace today, selecting a safe and effective app has become an urgent priority.

As health care regulators continue to struggle to effectively regulate health apps, clinicians and patients must make decisions today without formal support.^2^ Numerous app evaluation systems have arisen as a result and aim to provide direct recommendations or guidance to aid users in their selection of relevant apps. For example, a recent review identified at least 44 frameworks for assessing mental health apps^3^ and new ones are continually being introduced.^4^ Although each framework offers unique benefits, few offer concrete information about an app and are impractical for clinicians and patients to use.

Appreciating the need for actionable guidance around mental health apps via a scalable digital framework, our team has developed,^5^ introduced,^6^ and maintained^7^ the M-Health Index and Navigation Database (MIND). Derived from the principles behind the American Psychiatric Association’s (APA’s) app evaluation framework,^8^ MIND evaluates each app across 105 unique dimensions. These evaluation questions are designed to be clinically relevant, easy to update, as objective as possible, auditable, and searchable in a database to ensure any user can use the data to make an informed decision.

As a large public database of mental health apps, MIND offers a practical and data-driven approach for assessing the commercial app marketplace and trends. Since our team’s last examination of this data, based on March 2021 data,^6^ we have more than doubled the number of apps included in the database to now over 650 and maintained all app ratings up to date within 6 months of the last review. Using this unique data set, we can assess how the app marketplaces are responding to public needs.

As there have been more public-facing efforts revealing privacy flaws in many popular mental health apps^9,10^ and calls for apps to be more evidence-based,^11^ it remains unclear if there have been any changes in the mental health apps that most people may be downloading from the Apple App Store and Google Play Store. Thus, we assessed whether there is an association between popularity metrics and privacy scores for mental health apps.

Additionally, both patients and clinicians must understand what apps currently offer so that expectations around apps’ functionality, use cases, cost, and evidence can be realistic. For this reason, we analyzed and reported the origin and accessibility, privacy and security, clinical foundation, features and engagement styles, inputs and outputs, interoperability, apps supporting serious mental illness conditions, and passive data collection offered by all 578 apps.

In this cross-sectional study, the objective was to assess the current state of mental health apps, determine how or if the mental health app landscape has changed, and examine whether there is a correlation between app privacy scores and popularity as measured by star ratings and downloads. We hypothesized that the mental health app landscape would not have changed substantially since our previous analysis in 2021 and that there would be no correlation between app privacy scores and popularity metrics.

## Methods

This study follows the Strengthening the Reporting of Observational Studies in Epidemiology (STROBE) reporting guideline for cross-sectional studies. As this study did not involve human participation, there is no informed consent nor participant information. Instead, we provide app selection, criteria, and characteristics. Institutional review board review was not sought in accordance with 45 CFR §46.

Informed by the APA’s app evaluation framework,^12^ MIND is a publicly available database that involves a comprehensive assessment of mental health-related apps across 6 categories: (1) app origin and accessibility, (2) privacy and security, (3) clinical foundation, (4) features and engagement, (5) inputs and outputs, and (6) interoperability.

All apps in MIND were gathered from the Apple App Store or Google Play Store. Since the last evaluation of the database in 2021,^6^ apps supporting conditions for headache (n = 48), pain (n = 47), sleep (n = 106),^7^ eating disorders (n = 65),^13^ and substance use (smoking and tobacco) (n = 228)^14^ were added. Top mental health apps were evaluated using MIND, which involves answering 105 objective app questions. Apps were entered into MIND by 1 of 10 raters. App raters consisted of college students, medical students, and research assistants. Each rater underwent a 4-hour interrater reliability training based on our published methods.^5^ The training involved an online informational module, followed by practice rating at least 2 apps to assess initial reliability. Reliability was assessed using Cohen κ statistic,^15^ for which raters were required to demonstrate good interrater reliability as measured by a minimum threshold of 0.7. All raters surpassed this minimal threshold.

Following training, raters downloaded, examined, and entered answers to the 105 questions into MIND. Only apps that cost $10 or less to download were evaluated. No in-app purchases or subscriptions were made for evaluation. Raters used the basic app version, app store descriptions, free trials, and detailed list of features available through subscription or in-app purchases to evaluate apps.

### Statistical Analysis

#### Analysis of Descriptive Statistics and Correlation

Analysis was performed on apps available in the database as of June, 2022. We first determined the count and the proportion of apps in our database satisfying each MIND criterion. We then aimed to determine if pairs of MIND criteria were correlated with a statistically significant degree using Fisher exact test for independence. We were most interested in seeing whether there was a correlation between apps having various privacy settings and apps taking data from more sensitive input data sources, such as the smartphone’s microphone or GPS sensor. Correlational statistics were used since the variables of interest are all categorical. The threshold for statistical significance was set at 2-sided *P* < .05.

Additionally, between February and June 2022, app raters updated all 578 apps within 180 days of their last entry into the database. We analyzed the number of updates to each MIND criteria, as well as the total number of updates across the entire database.

#### Analysis of Privacy and Consumer-Facing Data

To quantify the privacy capabilities of each app in our database, we identified 5 MIND privacy criteria that we believed every mental health app should satisfy. These criteria included (1) having a privacy policy, (2) reporting security measures in place, (3) declaring data use and purpose, (4) allowing for the deletion of data, and (5) allowing users to opt out of data collection. Each app was assigned a score from 0 to 5, as the sum of these criteria.

We then analyzed the potential correlation between these underlying privacy scores based on objective empirical data and more consumer-facing measures of popularity, such as downloads. The Apple App Store does not provide the number of app downloads publicly. We scraped and collected this public data from app vendors using Python 3.8.8. The Google Play Store provides download data into categories: those with less than 50 000 downloads and those with 50 000 downloads or more. We ran a χ^2^ analysis on the resulting categorical data to calculate the correlation between downloads and our privacy scores. For the continuous rating data on both the Google Play Store and the Apple App Store, we used the *P* value associated with a Pearson correlation coefficient to determine statistical significance. Finally, we used a 2-sample *t* test to see if apps exclusively on the Google Play Store had a lower mean privacy score than apps exclusively on the Apple App Store.

## Results

### Descriptive Statistics

#### App Origin and Accessibility

Of the 578 apps included in this study, 160 (27.7%) were available only on iOS, 154 (26.6%) were available only on Android, and 264 (45.7%) were available on both iOS and Android. In addition to iOS and/or Android availability, one hundred of the 578 apps (17%) were also available on the web.

There were 525 apps (91%) developed by for-profit companies. Twenty-six (4%) were created from a government or governmental organization, 25 (4%) from a non-profit organization, 18 (3%) from a health care company, and 17 (3%) from an academic institution.

Although 507 apps (88%) were free to download, only 227 (39%) were completely free. Thus, many apps involved in-app purchases (44% [n = 254]) or subscriptions (34% [n = 194]) to unlock the entire app functionality.

All apps were available in English, and 105 (18%) were also available in Spanish. There were 378 apps (65%) that functioned offline in that they operated without connection to the internet, and 312 apps (54%) were identified as having at least one accessibility feature.

#### Privacy and Security

Of the 578 apps in the database, 443 (77%) had a privacy policy. Using the Flesch-Kincaid grade level readability formula, the mean (SD) reading grade level for these privacy policies was 12.5 (2.3). This indicates that the reader needs above a grade 12 reading comprehension level to understand the privacy policy. There were 257 apps (44%) that shared personal health information with third parties.

#### Clinical Foundation

Eighty-five apps (15%) offered either a feasibility or efficacy study. The quality of these studies was not assessed.

#### Features and Engagement

**Figure 1.**
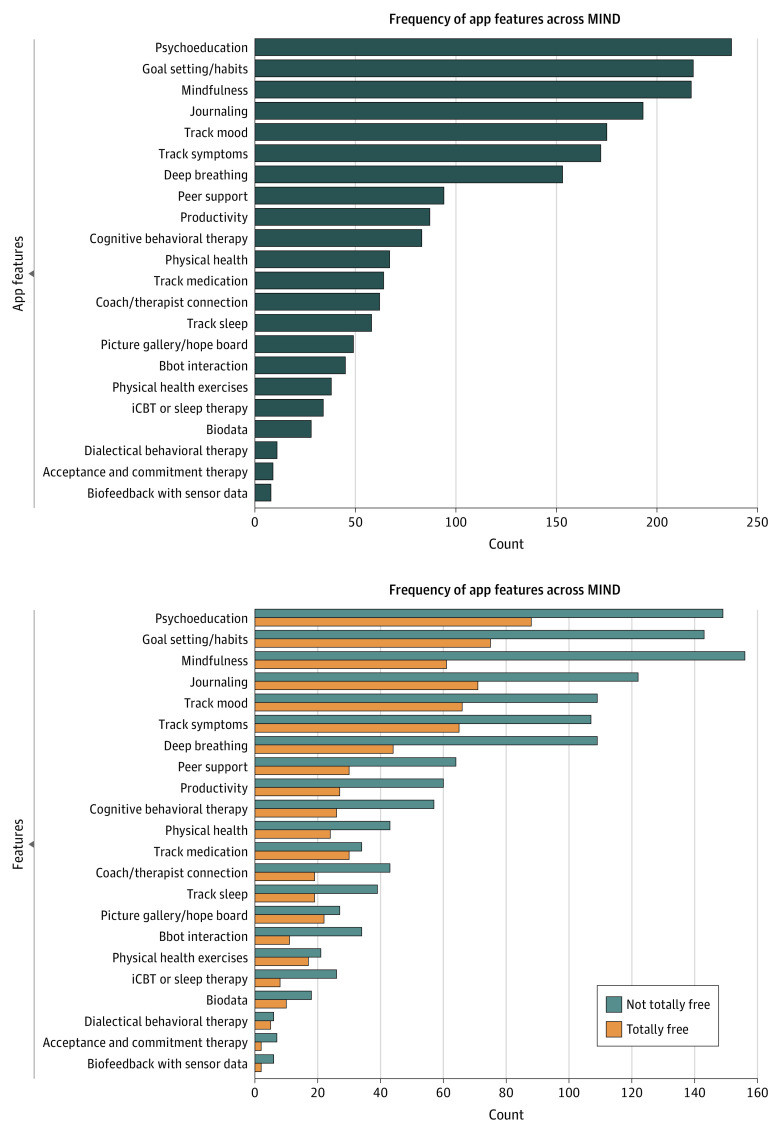
App Features Offered in M-Health Index and Navigation Database (MIND) From Most Common to Least Common iCBT indicates Cognitive Behavioral Therapy for Insomnia.

The apps examined offered 22 unique features related to therapeutic features. Of these, the most common feature was psychoeducation with 237 (41%), followed by goal setting/habit at 218 (38%) and mindfulness at 217 (38%), see Figure 1. Of note, the least common features were apps providing biofeedback with sensor data (1%), Acceptance and Commitment Therapy (2%), and Dialectical Behavioral Therapy (2%).

#### Inputs and Outputs

The most common inputs included surveys (45%), diary entries (34%), and microphone (21%). The most common outputs were notifications (68%), a summary of data (61%), and references and information (50%). Eighty-four apps (15%) collected passive data or data that was not entered into the app, specifically biodata such as step count and heart rate, or geolocation.

#### Interoperability

There were 172 apps (30%) in the database that allowed users to email or export their data. Only nine apps (2%) offer integration with an electronic medical record.

#### Apps for Serious Mental Illness

The 3 most common types of conditions our apps purported to help treat were substance abuse related to smoking or tobacco (33%), stress and anxiety (28%), and nonserious mood disorders (20%). By contrast, only 13 apps (2%) were built to address schizophrenia. Additionally, to a statistically significant degree, for-profit companies were disproportionately less likely to produce apps addressing serious mental illness.

#### Correlation Between Input Data Streams and Privacy

Apps which collected biodata, geolocation data, and accessed users’ cameras and microphones were more likely to implement privacy and security measures. A Fisher exact test found that all associated correlation statistics were statistically significant except for the correlation between collecting geolocation data and declaring data use and purpose (Table 1).

**Table 1.** Significant Correlations Between Sources of Input Data and Privacy Settings

	Correlation coefficient, *r*			
	Biodata	Geolocation	Camera	Microphone
Has privacy policy	0.0387	0.0192	<0.0001/	0.0006
App reports security measures in place	0.0096	0.0026	0.0001	0.0218
App declares data use and purpose	0.0058	NA	0.0018	0.0008
Can delete data	0.0495	0.0027	0.0035	0.0003
Is deidentified data shared	0.0003	<0.0001	<0.0001	0.0001

Abbreviation: NA, not applicable.

### Longitudinal Change

Between February and June 2022, 10 app raters updated all 578 apps within 180 days of their last entry into the database. A total of 371 apps (64%) had at least 1 change among the MIND criteria For detailed results, see the eResults in Supplement 1.

As compared with the 2021 results, we found that the number of apps collecting passive data, including biofeedback with sensor data, biodata, and geolocation, increased from 71 to 78 apps, despite the overall size of the analyzed sample more than doubling from 278 to 578 apps. We additionally noticed that the top 7 app features in 2021 remained the top features today, namely (1) mood tracking, (2) journaling, (3) mindfulness, (4) psychoeducation, (5) deep breathing, (6) symptom tracking, and (7) goal-setting and habits.

### Privacy and Consumer-Facing Data

#### Google Play Store Downloads

Of the 418 Android apps in MIND, 412 had data that we could successfully scrape regarding downloads on the Google Play Store. The number of app downloads on the Google Play Store, however, was correlated with privacy scores (χ^2^_5_ = 22.1; *P* < .001), see (Table 2). Thus, the more app downloads, the larger the privacy score.

**Table 2.** Android Apps Sorted by Privacy Score and Number of Downloads

	Android downloads, No. (%)		Total
	<50 000	≥50 000	
Privacy score (out of 5)			
0	58 (79)	15 (21)	73
1	9 (60)	6 (40)	15
2	25 (68)	12 (32)	37
3	67 (59)	46 (41)	113
4	59 (48)	65 (52)	124
5	25 (50)	25 (50)	50

#### Google Play Store Ratings

**Figure 2.**
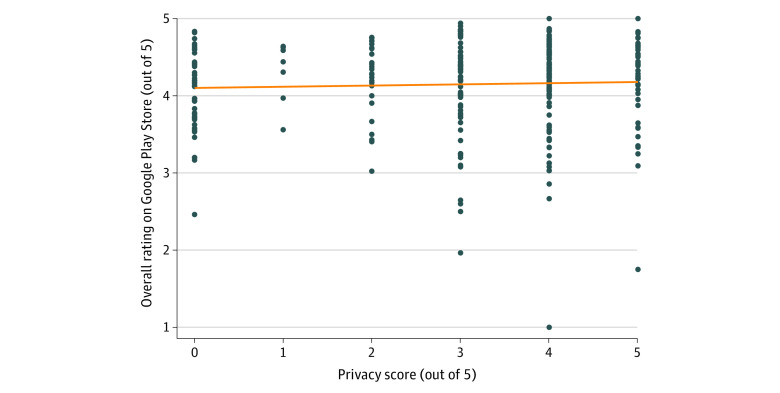
Comparison of Google Play Store Star Ratings and Privacy Scores (N = 305)

Of the 418 Android apps in our database, 305 had enough reviews from the public for the Google Play Store to display a dedicated ratings and reviews section with summary statistics such as the average star rating out of 5. These average and overall ratings on the Google Play Store were uncorrelated with our privacy scores (*r* = 0.041, *P* = .48) (Figure 2).

#### iOS App Store Ratings

**Figure 3.**
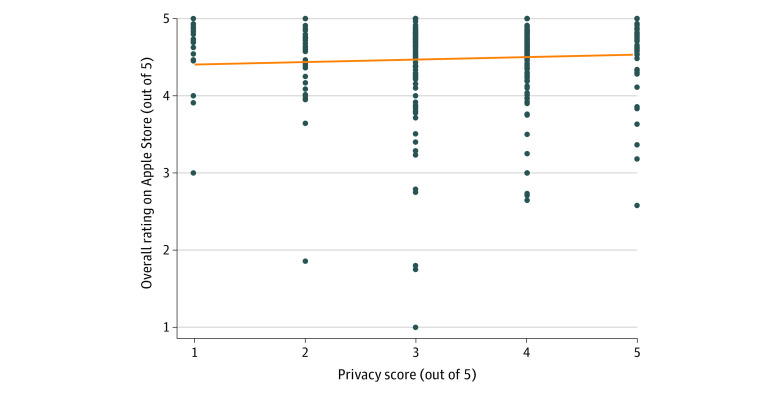
Comparison of Apple App Store Star Ratings and Privacy Scores (N = 340)

Apple imposed a policy requiring app developers to disclose privacy settings when they next update their apps. To assess the most up-to-date correlation between privacy scores and App Store ratings, we discarded apps that had not been updated recently enough to incur Apple’s new policy. The remaining 340 iOS apps had a privacy score of at least 1 because having a privacy policy was 1 of our 5 privacy criteria. There was no statistically significant correlation between App Store ratings and privacy scores (*r* = 0.058, *P* = .29) (Figure 3).

#### Google Play Store and iOS App Store Comparison

Of the 314 apps which were either on the Google Play Store or the iOS App Store but not both, there was not a statistically significant difference in mean privacy score (*t* = 1.9586, *P* = .051). See the eFigure in Supplement 1 for the distribution of privacy scores between Android and iOS apps.

## Discussion

We examined and assessed 578 mental health-related apps rated from January to July 2022 across 105 criteria per app using MIND. From a longitudinal perspective, we note that, since nearly two-thirds of apps featured at least 1 change in the 105 questions across 6 months, it is clear that the temporal dynamics of the marketplace present a challenge to traditional rating and scoring systems, and instead require consistent updating of mental health app databases.

While the potential of apps to increase the quality of and access to care remains high, the current commercial offerings face challenges. Leveraging new sensor and biological data to drive just-in-time adaptive interventions are often discussed, but we found that, within our sample, less than 5% of apps currently supported this capability. Another challenge seen within the mental health app marketplace is a lack of accessibility. Accessibility features may include adjustable text size, text-to-speech or speech-to-text abilities, and a colorblind color scheme adjuster. Only half of the apps analyzed included an accessibility feature. Related to access, our results echo the literature indicating the limited availability of mental health apps offered in Spanish,^16^ with only 18% of apps with Spanish language functionality. While all apps in the database are accessible to anyone, only 13 (2%) were designed to support individuals experiencing schizophrenia. These findings suggest that while the app marketplace offers a plethora of apps, marketplace forces alone are not creating an ecosystem of apps that are accessible to all or increasing access in patient groups that may need the most support.

Digital privacy, especially surrounding mental health apps, is a frequent topic of concern. Our results suggest ongoing privacy concerns among popular apps, with app marketplace star ratings providing no correlation with actual privacy features in place. But it is encouraging to see that 77% of apps reviewed possessed a privacy policy. However, with the mean (SD) grade reading level of these privacy policies at 12.5 (2.3), it remains unclear if these policies are accessible by most users. This result echoes many other results in the literature concerning barriers to privacy in digital mental health.^17,18,19^ Compared with our prior results in the 2021 analysis of MIND, we note that the grade reading level has not changed in more than a year^6^ and remains a barrier to understanding data privacy.

The lack of correlation between privacy scores and consumer ratings on both the iOS App Store and the Google Play Store also suggests consumers may not be aware of or seeking apps based on privacy features—underscoring an opportunity for further education or even regulation. A 2020 survey study conducted on data sharing revealed that only 9% of US consumers surveyed were inclined to share their personal health information with a tech company aiming to improve health care.^20^ Despite this, mobile mental health apps with data privacy issues remain popular, as seen in high star ratings and the number of downloads. Recently, the Mozilla Foundation assessed the privacy and security of 32 popular mental health and prayer apps and found that 87.5% of these apps posed substantial privacy concerns for users surrounding their data usage and management.^10^ The consistent privacy concerns among these apps suggest that new regulations may be necessary to enforce higher standards,^21^ alongside efforts to educate both patients and clinicians about the risks and benefits of these apps.

Overall, there is a lack of clinically supported mental health apps. A 2020 systematic search for wellness and stress-related apps available in app stores found that only 2 percent of their more than 1000 app samples had research studies to support app claims.^22^ Leong et al^23^ identified only 3.4% (6/179) of apps supporting anxiety and depression found in app stores with scientific evidence in the form of randomized clinical trials or real-world evidence.^23^ This situation on the app marketplaces parallels concerns regarding the lack of high-quality research studies on mental health apps^24^ and highlights an opportunity for industry and academic collaboration.

Our findings also offer positive interpretations. With so many apps on the commercial marketplaces, clinicians and patients can now be more demanding in what they seek from any given app. Since many apps share similar features (Figure 1), searching for apps that offer that feature for free while also protecting privacy is reasonable. With 39% of apps examined still completely free, and free apps offering similar privacy protections and levels of evidence as paid apps, turning to free apps first may be recommended.

### Limitations

As with all studies, our methods have limitations. Our data are derived from app self-declaration and examination of apps based on the MIND questions. We did not evaluate the quality of different app features or the quality of the science underlying these apps. This is a nearly impossible task given the rapid turnover and changes of these apps compared with the time, effort, and expense of conducting a technical audit of each app. We also only examined apps that cost $10 or less. Thus, our sample may not be indicative of the entire app landscape as these more costly apps may offer different properties. However, since we analyzed the top apps across various conditions from both app stores, our findings may be indicative of the landscape for the most popular and widely used mental health apps. Lastly, in this study, the content type was collected and analyzed, whereas content quality was not. Recent research highlights the difficulty in assessing study quality for mobile mental health apps due to heterogeneity.^24^

## Conclusions

The findings of this cross-sectional study suggest that the current app marketplaces lack diversity in their offerings and fail to implement potentially high-impact features. Another challenge to the app space is that easily accessible metrics like star ratings fail to consider privacy capabilities. Thus, clinicians and patients must discern apps beyond such measures to ensure the discovery of apps that both fit their unique needs and protect their privacy. Publicly available app libraries^25^ and validated app evaluation frameworks like MIND are innovative tools to support users in their app selection.

## Notes

Supplement 1. **eResults.** Change in Mental Health Apps After 180 Days Since Their Last Entry in MIND **eFigure.** Comparison of Privacy Scores Between Android and iOS Applications

Supplement 2.
Data Sharing Statement
