# Supplementary material for: Assessment of Mental Health Services Available Through Smartphone Apps
Source: JAMA Netw Open. 2022 Dec 28;5(12):e2248784. doi: 10.1001/jamanetworkopen.2022.48784 (PMC9857226; doi:10.1001/jamanetworkopen.2022.48784)
Supplement: Supplement 1. — eResults. Change in Mental Health Apps After 180 Days Since Their Last Entry in MIND eFigure. Comparison of Privacy Scores Between Android and iOS Applications [file jamanetwopen-e2248784-s001.pdf]

Supplemental Online Content

Camacho E, Cohen A, Torous J. Assessment of mental health services available through smartphone apps. *JAMA Netw Open.* 2022;5(12):e2248784. doi:10.1001/jamanetworkopen.2022.48784

eResults. Change in Mental Health Apps After 180 Days Since Their Last Entry in MIND

eFigure. Comparison of Privacy Scores Between Android and iOS Applications

This supplemental material has been provided by the authors to give readers additional information about their work.

© 2022 Camacho E et al. *JAMA Network Open.*

eResults. Change in Mental Health Apps After 180 Days Since Their Last Entry in MIND

Longitudinal Change:

Days Since Last Update

We gathered how many days it had been since the apps were last updated. Ten of the 578 apps have not been updated since development. The five-number summary for the number of days since each of the 568 apps were updated on the Apple app store or Google play store is Min: 12, Q1: 132, Med: 381, Q3: 972, Max: 3210.

App Origin and Accessibility

App cost structure changed in 76 apps (13%), with 13 of these app changes involving removal of a one-time purchase to download. 58 apps added an accessibility feature, 8 apps became available in Spanish, and 11 apps became available offline or without the use of internet.

Privacy and Security

The most common privacy-related change was increased privacy policy reading grade level, with 25 of the 443 apps with privacy policies identified with this change. However, 20 apps saw a decrease in privacy policy reading grade level. 22 apps removed their privacy policy altogether.

Clinical Foundation

The most frequently observed changes in clinical foundation were the addition of supporting studies (n=19), the addition of a use warning that the app is not a replacement for care (n=14), and the addition of potential for harm (n=8).

Features and Engagement

The most common feature additions were goal setting (n=15), psychoeducation (n=11), and symptom tracking (n=10). The leading engagement style additions included videos (n=14), asynchronous response (n=9), and peer support (n=8).

Inputs and Outputs

The primary changes in inputs involved the addition of microphone usage (n=41), logging in through a social network (n=16), and survey collection (n=15). The most common output additions were the ability to post from the app to your social media (n=31), reminders (n=17), and notifications (n=14).

Interoperability

Twelve apps added the ability to export user data and one app added the ability to integrate into an electronic medical record.

© 2022 Camacho E et al. *JAMA Network Open.*

eFigure. Comparison of Privacy Scores Between Android and iOS Applications

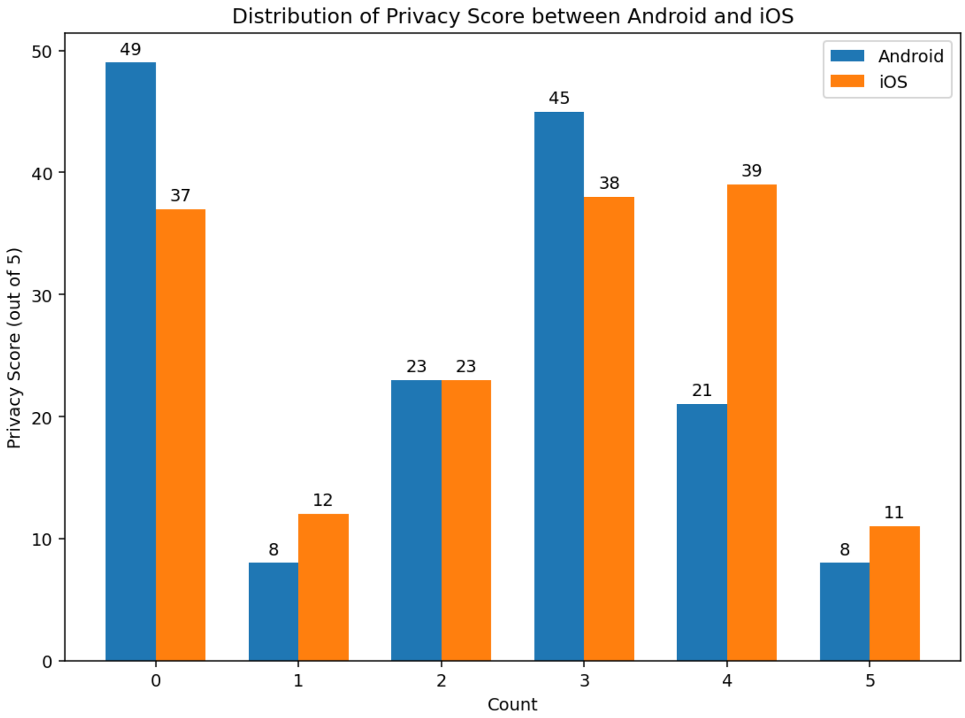

© 2022 Camacho E et al. *JAMA Network Open.*
