# Supplementary material for: Assessment of Mental Health Services Available Through Smartphone Apps
Source: JAMA Netw Open. 2022 Dec 28;5(12):e2248784. doi: 10.1001/jamanetworkopen.2022.48784 (PMC9857226; doi:10.1001/jamanetworkopen.2022.48784)
Supplement: Supplement 2. — Data Sharing Statement [file jamanetwopen-e2248784-s002.pdf]

# Data Sharing Statement

Camacho. Assessment of Mental Health Services Available Through Smartphone Apps. *JAMA Netw Open*. Published December 28, 2022. doi:10.1001/jamanetworkopen.2022.48784

**Data**
**Data available:** Yes
**Data types:** Data (not involving human participants), Data dictionary
**How to access data:** https://mindapps.org/Home
**When available:** With publication

**Supporting Documents**
**Document types:** None

**Additional Information**
**Who can access the data:** https://mindapps.org/Home is accessible to all people at anytime
**Types of analyses:** The data presented is open to all uses.
**Mechanisms of data availability:** Without investigator support
